# Printed Nanomaterials for All-in-One Integrated Flexible Wearables and Bioelectronics

**DOI:** 10.1021/acsami.4c17939

**Published:** 2024-11-25

**Authors:** Youngjin Kwon, Jongsu Kim, Hojoong Kim, Tae Woog Kang, Jimin Lee, Seung Soon Jang, Yongkuk Lee, Woon-Hong Yeo

**Affiliations:** † Wearable Intelligent Systems and Healthcare Center (WISH Center), Institute for Matter and Systems, Georgia Institute of Technology, Atlanta, Georgia 30332, United States; ‡ School of Materials Science and Engineering, Georgia Institute of Technology, Atlanta, Georgia 30332, United States; § George W. Woodruff School of Mechanical Engineering, Georgia Institute of Technology, Atlanta, Georgia 30332, United States; ∥ Korea KIAT-Georgia Tech Semiconductor Electronics Center (K-GTSEC), Georgia Institute of Technology, Atlanta, Georgia 30332, United States; ⊥ Department of Biomedical Engineering, Wichita State University, Wichita, Kansas 67260, United States; ¶ Wallace H. Coulter Department of Biomedical Engineering, Georgia Institute of Technology and Emory University School of Medicine, Atlanta, Georgia 30332, United States; ∇ Parker H. Petit Institute for Bioengineering and Biosciences, Georgia Institute of Technology, Atlanta, Georgia 30332, United States

**Keywords:** nanomaterials, aerosol-jet printing, flexible electronics, wearables, bioelectronics

## Abstract

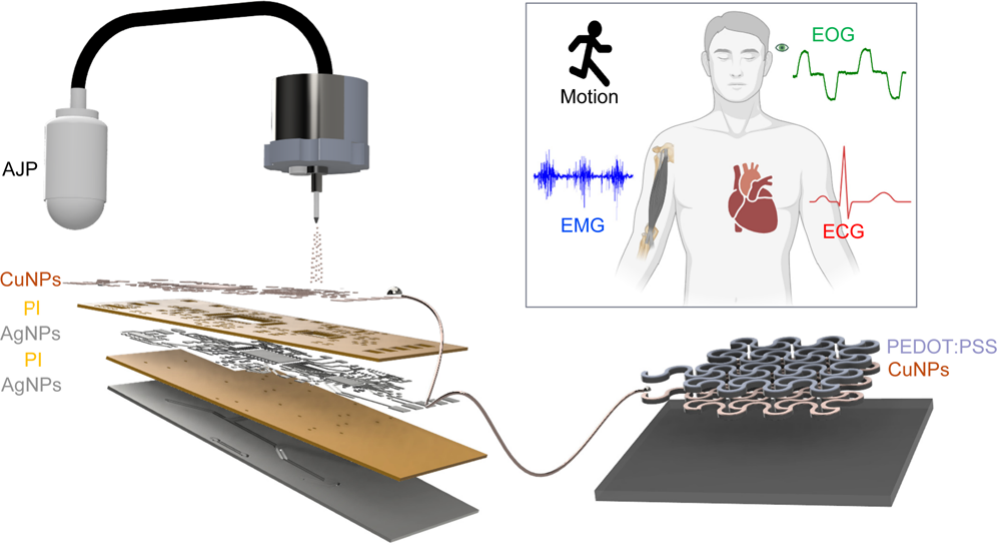

Recent advancements in printing technologies allow for fabricating various wearable sensors, circuits, and integrated electronics. However, most printing tools have limited ranges of handling ink viscosity, a short working distance, and a limited feature size for developing sophisticated electronics. Here, this paper introduces an all-in-one integrated wearable electronic system via multilayer, multinanomaterial printing. Versatile, high-resolution aerosol-jet printing could successfully print Cu nanoparticles, Ag nanoparticles, PEDOT:PSS, and polyimide (PI) to manufacture nanomembrane composite structures, including skin-contact electrodes and wireless circuits. The printed polymer, PEDOT:PSS deposited on the Cu, ensures biocompatibility when making direct skin contact while enhancing electrical conductivity for electrodes. A self-assembled monolayer facilitates better adhesion of Cu nanoparticles on the PI. Also, using intensive pulsed light, a photonic sintering method minimizes Cu-oxidation while avoiding thermal damage. The combined experimental and computational study shows the mechanical flexibility and reliability of the printed integrated device. With human subjects, the flexible wireless bioelectronic system demonstrates superior performance in detecting high-fidelity physiological signals on the skin, including electromyograms, electrooculograms, electrocardiograms, and motions, proving its potential applications in portable human healthcare and persistent human–machine interfaces.

## Introduction

Various printing technologies have significantly impacted the development of flexible electronics, particularly in the realm of biosensors. Different printing techniques, such as inkjet printing (IJP), screen printing (SP), 3D printing (3DP), meniscus-guided printing (MGP), and aerosol-jet printing (AJP), have been utilized to fabricate electrodes and circuits for biosensing applications.^1−7^ These methods offer the ability to create lightweight, flexible, and skin-conformal devices, enabling the monitoring of physiological signals, including electromyograms (EMG), electrocardiograms (ECG), electrooculograms (EOG), and electroencephalograms (EEG).^8−15^ Among these, AJP stands out due to its high-resolution patterning, scalability, and reduced material waste, making it an attractive choice for creating complex, multilayered structures directly onto various substrates.^2,16^ Compared to other printing techniques, AJP can handle a wide range of ink viscosities (from 1 to 1000 cP) without the need for masks or screens, providing versatility in fabricating flexible and wearable devices.^17−20^ However, while many studies have used different printing techniques to form electrodes and circuits, it is rare to find research that combines both elements in a single study to detect multiple physiological signals using a comprehensive approach (Table 1).^1,5−7,12,14−16,21−25^

Furthermore, most existing work relies on expensive materials such as Ag, graphene, and liquid metals. While graphene has been utilized for its biocompatibility and solderability, it often exhibits limited electrical and mechanical properties, resulting in suboptimal performance for certain applications (Table 2).^1^ In contrast, Cu presents a cost-effective alternative with significantly higher electrical conductivity and mechanical robustness, making it a promising candidate for printed electronics.^1,11,26−28^ However, Cu presents several challenges, particularly its tendency to oxidize, biocompatibility issues, and susceptibility to corrosion from substances such as sweat.^2,29−34^ To address these challenges, our work employs the synthesis of Cu inks using PVP and NaH_2_PO_2_–H_2_O, a well-established method, in conjunction with AJP. This combination allows for the fabrication of complex, multilayered electrodes and circuits with enhanced precision and scalability, which has not been explored in prior research. Additionally, by carefully controlling the particle size distribution of the Cu nanoparticles, which remain well-dispersed and stable over time, we ensure improved printability and consistent electrical performance. Another major issue with Cu is its susceptibility to thermal damage due to the thermal expansion coefficient mismatch with flexible substrates.^17,19,20,32,35−37^ These problems have limited its widespread use in printed electronics. To address these issues, lower-temperature sintering methods, such as intense pulsed light (IPL) sintering, have been explored to minimize thermal damage.^33,35,38^ However, despite these advances, additional protective strategies, such as applying PEDOT:PSS as a capping layer, are necessary to prevent oxidation and improve the biocompatibility of Cu electrodes.

**Table 1:** Comparison of Various Printed Wearable Devices for Detecting Physiological Signals^a^

reference	fabrication method	electrode material	circuit material	measured signal
this work	AJP (electrode, circuit)	PEDOT:PSS/CuNPs/PI	CuNPs/Ag/PI	EMG, ECG, EOG, motions
(6)	SP	Ag/elastomer		EMG
(22)	SP	Ag/AgCl		ECG
(14)	AJP (electrode only)	Ag/PI	Cu/PI	EOG
(1)	AJP (electrode, circuit)	graphene/PI	graphene/Ag/PI	EMG
(23)	IJP	AgNPs		EMG
(24)	AJP	AgNPs		EMG
(15)	SP	PI/PEG		ECG
(25)	3DP	Cu/Ga		PPG
(7)	MGP	Ga–In alloy		ECG, EMG
(51)	AJP	AgNPs		EEG
(5)	3DP	CNT/PDMS		EEG, EOG

a AJP: aerosol-jet printing. SP: screen printing. IJP: inkjet printing. 3DP: 3D printing. MGP: meniscus-guided printing.

**Table 2:** Comparison of Various Properties of CuNP and Graphene for Bioelectronic Applications

	CuNP	graphene	references
price	cheap	expensive	(52,53,54,55)
sheet resistance	4.2 Ω/sq	1.5 kΩ/sq	(56)
resistivity	11.72 μΩ·cm	1.4–3 mΩ·cm	(57,58)
solderability	O	O	(1)
skin impedance	11.5 kΩ	97.1 kΩ	(1)
SNR	38.59 dB	97.1 dB	(1)
*R*/*R*_0_ change under 60% stretching	1–1.002	1–1.25	(1)
biocompatibility	X	O	(1)

Here, this paper studies various nanomaterials, SAM treatments, IPL sintering, and AJP methods for developing fully printed, all-in-one, integrated, flexible electronics. We focus on integrating multiple materials to fabricate a sensor-integrated wireless system to measure various physiological signals on the human skin. With optimization, we can successfully print various inks, such as CuNPs, AgNPs, PEDOT:PSS, and PI, to develop flexible membrane electrodes and circuits. To address Cu’s inherent limitations and achieve lower resistivity, we explore self-assembled monolayers, which enhance the adhesion of CuNPs, and employ IPL photonic sintering to minimize thermal damage during sintering. Additionally, PEDOT:PSS is deposited on CuNPs to prevent oxidation and ensure biocompatibility, making them suitable for long-term physiological monitoring. Compared to other printing techniques, the comprehensive method presented in this work offers to fabricate more cost-effective but high-performance wireless wearable electronics. Demonstrating the device’s performance with human subjects shows the versatility of the fabricated all-in-one wireless system in detecting various physiological signals, such as EMG, ECG, EOG, and motions; the signal quality from this single device platform is as good as the clinical-grade devices using bulky electronics and rigid sensors. Collectively, this study using printing methods and various nanomaterials opens up opportunities to use the integrated wearable platform when targeting portable human health monitoring, continuous disease diagnosis, and therapeutics by combining sensors with actuators and rehabilitation tools.

## Results and Discussion

### Overview of an All-in-One, Printed, Wireless, Wearable Electronic System

**Figure 1:**
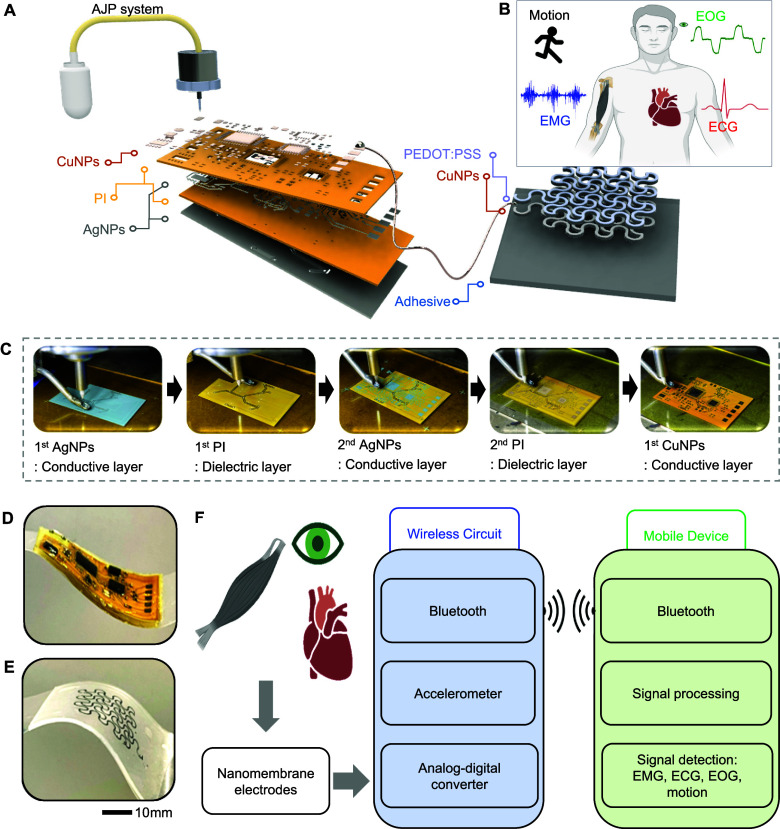
Overview of an all-in-one, printed, wireless, wearable electronic system for detecting multiple physiological signals. (A) AJP-based fabrication of integrated electronics using PI, AgNPs, CuNPs, and PEDOT:PSS for a multilayered circuit and an electrode. (B) Target physiological signals, measured by the fabricated wearable electronics, including EMG, ECG, EOG, and motion (created in BioRender). (C) Sequential photos showing a circuit printing process using five different layers with three materials. (D,E) Photos of a printed flexible circuit (D) and a printed stretchable electrode (E,F) flowchart showing the overall processes of signal detection, starting from electrode mounting on targeted skin to wireless data transfer with a circuit and data processing on a mobile device (created in BioRender).

Figure 1A shows an overview of the system, which includes printed stretchable electrodes and printed flexible circuits. The multilayered circuit comprises AgNPs, PI, and CuNPs, where the PI works as an insulating layer to package the metal layers. For the electrodes, CuNPs and PEDOT:PSS inks are printed on an adhesive substrate to directly attach to the human skin and measure physiological signals, such as EMG, ECG, EOG, and motion (Figure 1B). Figure 1C shows consecutive photos of the printing process for the flexible circuit. AgNPs, CuNPs, and PI inks are sequentially printed on a glass slide. The detailed step-by-step nanomanufacturing process is described in the Experimental Section. Figure 1D,E display close-up images of a fabricated flexible circuit (Figure 1D) and a stretchable electrode (Figure 1E) mounted on an elastomeric membrane. These mechanically compliant devices are designed to withstand prolonged wearability during signal measurement and to ensure accurate biopotential detection through close contact with the body. The soft elastomer facilitates conformal adhesion of the nanomembrane electrode to the skin. Figure 1F illustrates the data processing workflow of the printed wearable device, which wirelessly stores and displays various signals measured from various locations of the human body.

### Characterization, Photonic Sintering, and Surface Functionalization of CuNPs

**Figure 2:**
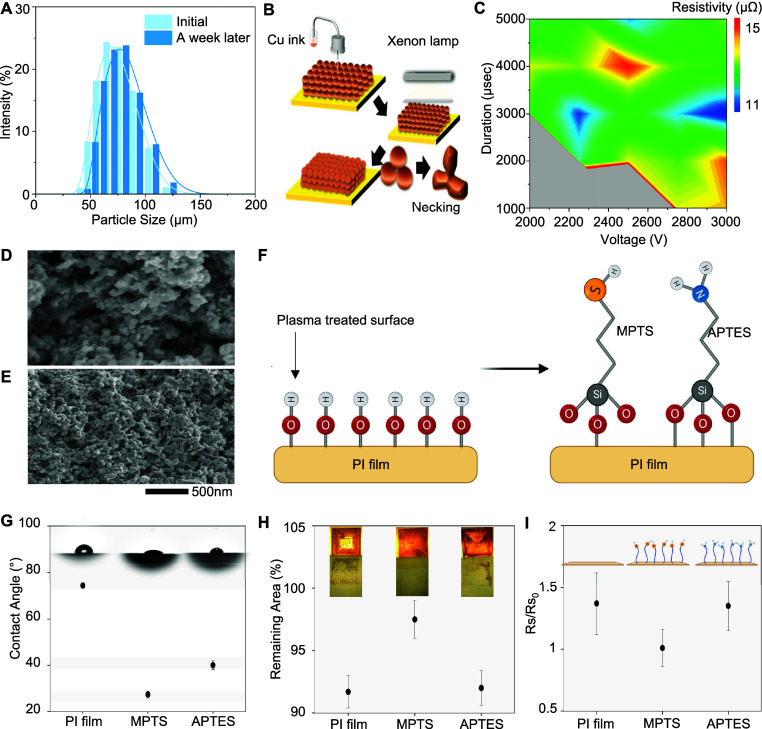
Photonic sintering and surface functionalization processes. (A) Particle size distribution of PVP-capped Cu NPs showing stable dispersion with an average size of 70 nm after preparation and storage for several days. (B) Illustration of the lamp-based photonic sintering process. (C) Electrical resistivity of IPL sintered CuNPs with various voltage and duration. (D,E) Comparison of scanning electron microscope images of two examples: an example of sintered CuNPs in low voltage and duration (D) and an example of sintered CuNPs in optimized voltage and duration (E). (F) Schematic illustration of the SAM surface treatment process using (3-mercaptopropyl)trimethoxysilane (MPTS) and (3-aminopropyl)trimethoxysilane (APTES) (created in BioRender), (G) measured contact angle data of the CuNP-printed substrate comparing untreated PI film, MPTS-treated, and APTES-treated surfaces, demonstrating improved wettability, with MPTS showing the most significant reduction in contact angle. (H) Comparison of the remaining area of CuNPs on PI film after the adhesion test for PI film, MPTS-treated, and APTES-treated surfaces. (I) Comparison of *R*_s_/*R*_s0_ (normalized resistance) after the adhesion test for PI, MPTS-treated, and APTES-treated samples (created in BioRender).

In this study, we characterize CuNP inks to ensure their suitability for making devices. As shown in Figure 2, the CuNPs are well dispersed with an average size of approximately 70 nm. This stable dispersion is primarily attributed to the use of PVP (polyvinylpyrrolidone) as a capping agent. PVP forms a protective layer around the nanoparticles, preventing aggregation through steric hindrance and stabilizing the particles in the aqueous solution. As demonstrated in Figure 2A, the particle size distribution remains stable over a one-week period, confirming that the PVP effectively maintains the dispersion quality without significant aggregation. This is consistent with previous studies, which highlight PVP’s role in maintaining uniform particle size distribution over time due to its ability to form hydration layers that stabilize nanoparticles even under extended storage conditions.^39^ Additionally, XPS analysis in Figure S1 confirms that the particles are composed of pure Cu, with no significant oxidation observed. The XPS spectra did not reveal any noticeable peaks corresponding to copper oxides, further verifying the absence of oxidation during both synthesis and storage. To ensure that the ink remains well-dispersed before each experiment, we subjected the CuNP solution to ultrasonication for 30 min, which further enhances particle uniformity and prevents agglomeration. These results demonstrate that the CuNP ink, stabilized by PVP, exhibits excellent stability and performance characteristics suitable for high-quality printing and sintering in our experiments. The preparation of CuNP ink involves dispersing CuNPs in deionized water to create a stable suspension, as illustrated in Figure S2. The detailed steps of this process are elaborated on in the Experimental Section. After preparation, the CuNPs are then printed using AJP and subsequently sintered at room temperature using IPL with a xenon lamp (Figure 2B). To find the optimal sintering conditions, various lamp voltages ranging from 2 to 3 kV and durations ranging from 1 to 5 ms are evaluated, aiming for minimal resistivity. As shown in Figure 2C, the optimal condition at 3 kV and 3 ms shows the lowest resistivity of 11.72 μΩ·cm. Although the measured resistivity is higher than the resistivity of bulk copper (1.72 μΩ·cm), using low-temperature IPL sintering is sufficient to achieve satisfactory performance as an electrode. The process effectively balances lower processing temperatures with adequate electrical conductivity, making it suitable for wearable electronics applications. Figure 2D shows a scanning electron microscope (SEM) image of not fully sintered CuNPs, while Figure 2E shows fully sintered CuNPs, demonstrating a high-quality film due to the optimized sintering conditions. The solderability of the printed and sintered CuNPs is further demonstrated in Figure S3, where the Cu surface successfully accommodates soldering, indicating its practical application for circuit assembly. To enhance the ink’s performance, we investigated two types of self-assembled monolayers (SAMs): (3-mercaptopropyl)trimethoxysilane (MPTS) and (3-aminopropyl)trimethoxysilane (APTES), as shown in Figure 2F, which compares the contact angles of the ink-printed substrates treated with each SAM.^29,40−42^ Both SAMs significantly improve the ink’s contact angle and surface energy (Figure 2G), with MPTS reducing the contact angle from 74.5° to 27.5° and APTES achieving approximately 40.1°. However, zeta potential data indicate that sulfur-containing MPTS bonds more effectively with copper due to the strong metal–thiolate interaction, as copper is slightly negative after AJP, allowing better interaction with sulfur. In contrast, the amine group of APTES, with its near–neutral interaction with copper, leads to weaker adhesion. As a result, MPTS could provide superior stability (Figure S4) where the sulfur group exhibits stronger adhesion compared to the amine group.^29,43^Figure 2H compares the adhesion results using the ASTM D3359 Cross Hatch Adhesion Test, showing that MPTS-treated samples have a remaining area of about 97.5%, while APTES-treated samples show a remaining area of 92.0%, slightly better than the untreated PI substrates (remaining area: 91.7%).^40,44^Figure 2I presents the change in resistivity (*R*_s_) after the adhesion test, with *R*_s_/*R*_s0_ ratios of 1.03 for MPTS-treated samples, 1.35 for APTES-treated samples, and 1.37 for untreated PI substrates. This result indicates that MPTS significantly improves adhesion and better maintains conductivity compared to APTES and untreated samples. By integrating the SAM treatments with IPL sintering, we can fabricate high-conductivity films and improve the overall performance of the CuNP ink, making it suitable for flexible wearable electronics.

### Characterization of Printed Electrodes and Circuits

**Figure 3:**
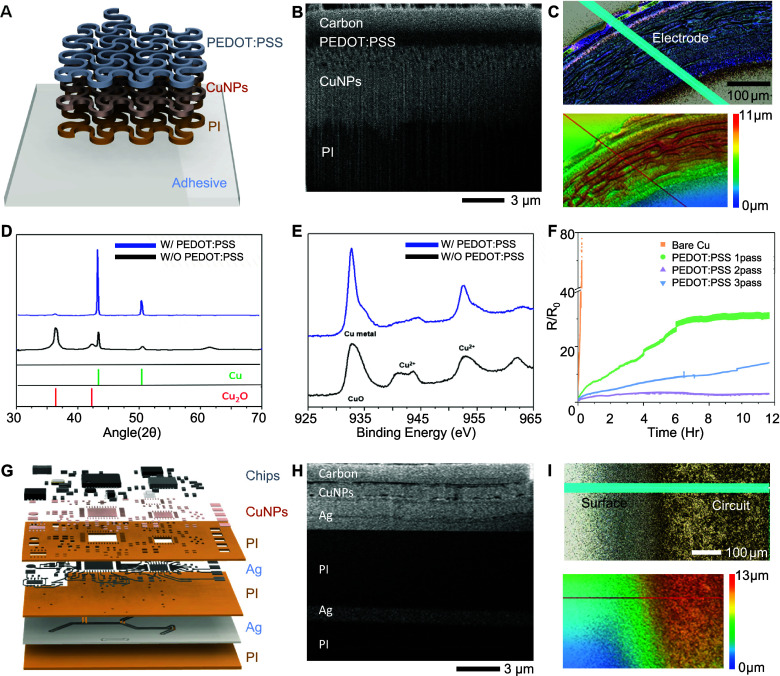
Characterization of printed electrodes and circuits. (A) Schematic illustration of the multilayered printed electrode. (B) FIB-assisted SEM image showing a cross-section of the printed electrode. (C) Photo and illustration of the measured cross-sectional profile of the electrode. (D) Result of XRD data showing the difference between with and without the PEDOT:PSS layer. (E) XPS data of sintered Cu and PEDOT:PSS in the printed electrode. (F) Resistance changes of Cu electrodes with different layers of PEDOT:PSS capping in artificial sweat over time. (G) Schematic illustration of the multilayered printed circuit with integrated chips, (H) FIB-assisted SEM image showing a cross-section of the printed circuit. (I) Photo and illustration of the measured cross-sectional profile of the printed circuit.

For this study, ultrathin and multilayered stretchable electrodes and flexible circuits are printed using AJP, as summarized in Figure 3. AJP offers two atomizing modes: ultrasonic and pneumatic. The ultrasonic mode allows printing inks with viscosities from 1 to 30 cP, while the pneumatic mode can manage viscosities between 1 and 1000 cP. In this study, the atomization and sheath gas streams for each ink have been experimentally optimized to ensure precise ejection of atomized droplets from the nozzle to the substrate. The printed electrodes include precisely aligned PEDOT:PSS, CuNPs, and PI layers printed with serpentine patterns (Figure 3A). These layers serve as antioxidant, sensing, and structural support layers, respectively. The total thickness of the printed electrodes is approximately 11 μm, including a 2 μm-thick PEDOT:PSS layer, a 6 μm-thick CuNPs layer, and a 3 μm-thick PI layer (Figure 3B,C). The thick PI layer contributes to the mechanical stability of the printed electrodes. Although CuNPs offer excellent electrical performance and are cost-effective, they present challenges when used as electrodes that come into contact with human skin. In particular, copper has biocompatibility issues and is prone to oxidation in air, as well as corrosion from substances such as sweat that are present on the skin.^2,29−34^ To address these problems, we used PEDOT:PSS, a biocompatible and conductive polymer, as a capping material. The PEDOT:PSS layer not only protects the CuNPs from oxidation and corrosion but also improves the overall biocompatibility of the electrodes, making them more suitable for wearable bioelectronic applications.^27^ However, PEDOT:PSS is generally deposited via spin-coating and patterned using conventional lithography, which increases fabrication cost and time.^28^ Forming a PEDOT:PSS/CuNPs bilayer using AJP reduces fabrication cost and time while providing high electrical conductivity and biocompatibility for long-term use (Figure 3D). This protection mechanism is illustrated in Figure S5, which shows a schematic representation of the oxidation process: bare Cu electrodes tend to form CuO over time, while Cu/PEDOT:PSS electrodes exhibit significantly reduced oxidation, effectively preventing the formation of copper oxide. X-ray diffraction (XRD) measurements highlight the critical role of the printed PEDOT:PSS layer (Figure 3E). The CuNPs layer without PEDOT:PSS layer peaks at 36.5° and 42.4°, suggesting CuNPs are oxidized to Cu_2_O. In contrast, the PEDOT:PSS/CuNPs bilayer only shows peaks at 43.8° and 50.8°, indicating a pure copper structure. X-ray photoelectron spectroscopy (XPS) analysis further supports this observation, showing small shakeup peaks around 940 eV in the absence of PEDOT:PSS, indicative of CuNPs oxidation (Figure 3F).^31,32^ To further investigate the protective effect of PEDOT:PSS on Cu electrodes, we conducted accelerated corrosion tests by immersing the printed electrodes in artificial sweat.^45^ This test aims to simulate real-world conditions where the electrodes would be exposed to sweat during prolonged skin contact. We prepared several samples, including uncoated Cu electrodes andCu electrodes coated with one, two, or three layers of PEDOT:PSS. The samples were then submerged in the artificial sweat solution, and the change in electrical resistance was monitored over time to evaluate the stability and durability of the electrodes. As shown in Figure 3F, the uncoated Cu electrodes show a significant increase in resistance within a brief period, indicating rapid oxidation and corrosion. In contrast, the PEDOT:PSS samples demonstrated much better stability. Specifically, the electrodes with two layers of PEDOT:PSS maintain their original resistance for the longest duration, suggesting optimal protection against corrosion. Interestingly, the samples with three layers of PEDOT:PSS show faster increases in resistance compared to the two-layer samples. This phenomenon may be attributed to the formation of internal stress and microcracks in the thicker PEDOT:PSS layers, as thicker films are more prone to crack formation during drying and thermal processes, which has been observed in prior studies of PEDOT:PSS.^46^ These cracks can compromise the protective barrier and lead to faster oxidation and corrosion. Table 3 and Figure S6 present the measured thickness of the Cu electrodes as a function of the number of PEDOT:PSS coating layers, showing a gradual increase in thickness with additional layers, which correlates with the observed trends in corrosion resistance. These results indicate that a two-layer PEDOT:PSS coating provides the best balance between corrosion protection and electrode performance, making it an effective strategy for enhancing the long-term stability and biocompatibility of printed Cu electrodes in wearable bioelectronics. Figure 3G illustrates the printed multilayered circuit using AgNP and CuNP inks as high-conductivity layers and PI ink as supporting and intermediate layers to prevent conductive shorts between metal layers. For flexible circuits, four layers of inks are printed, including AgNP, PI, AgNP, PI, and CuNPs. The first conductive layer, AgNPs with xylene, is deposited on a PMMA/PI-coated substrate using the ultrasonic mode. The first PI layer, printed on the sintered AgNP layer, works as a dielectric layer. The PI ink, a 3.5:1 mixture of PI (PI-2545) and 1-methyl-2-pyrrolidinone (NMP) has a lowered viscosity of 350 cP to be printable. Note that this PI layer was carefully printed so as not to clog the VIAs, allowing the first and second AgNP layers to connect. The second AgNP layer contains main circuit traces, while the second PI layer masks the second AgNP layer except for solder regions where functional chip components are mounted. As the last step, the CuNP ink is deposited only on solder regions of the second AgNP layer for solderability with solder paste. Then, the printed CuNP ink is sintered with low photonic energy (at 3 kV for 3 ms) to prevent oxidation before soldering. The total thickness of the printed circuit is approximately 10 μm, including 0.85 μm-thick first AgNP, 6.0 μm-thick first PI, 0.85 μm-thick second AgNPs, and 1.8 μm-thick CuNPs layers (Figure 3H,I). After printing all the layers, we integrated functional chip components via soldering, and the finalized device was encapsulated with a low-modulus silicone elastomer.

**Table 3:** Comparison of Oxidation Rates based on the Thickness of Cu/PEDOT Electrodes

layer		0	1	2	3
thickness		5.7	6.9	7.9	8.7
oxidation rate	Cu	9.3	12.24	32.29	16.97
	Cu_2_O	39.76	35.96	30.49	39.75
	CuO	50.94	51.8	37.22	43.28

### Mechanical Characteristics of Nanomembrane Electrodes and Circuits

**Figure 4:**
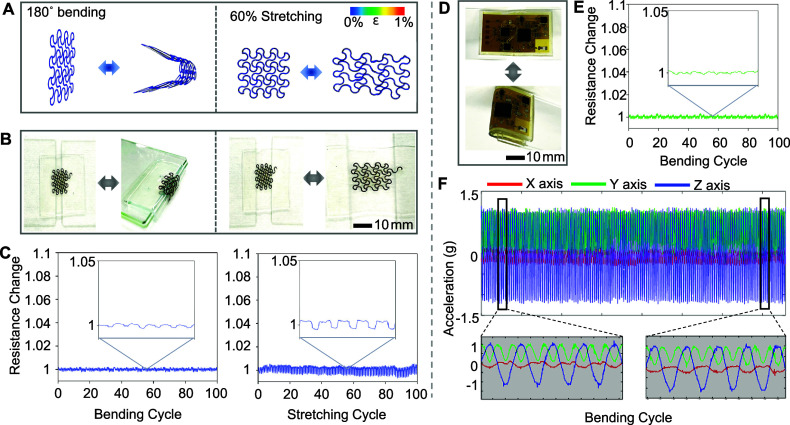
Mechanical characteristics of nanomembrane electrodes and circuits. (A) FEA results in designing mechanically stable electrodes with applied bending and tensile strain. (B) Experimental validation results of a fabricated electrode showing mechanical stability during bending and stretching. (C) Measured resistance changes of the fabricated electrode during 100 bending cycles (left) and 100 stretching cycles (right), showing negligible changes. (D) Photos of a fabricated circuit showing 180-degree bending. (E) Measured resistance changes of the fabricated circuit during 100 bending cycles. (F) Wirelessly measured 3-axis acceleration from the circuit obtained during 100 bending cycles, showing the device’s consistent performance in wireless connection and data acquisition.

Figure 4 summarizes computational and experimental studies on the mechanical flexibility, stretchability, and reliability of printed electrodes and circuits. Figure 4A shows FEA study outcomes in designing stretchable electrodes that can endure cyclic bending with a 1.5 mm radius and stretching (60% uniaxial). Based on the modeling, a set of electrodes has been printed and experimentally validated for mechanical stability during bending and stretching (Figure 4B), showing no visual damage. For quantitative analysis, Figure 4C summarizes measured resistance changes of the fabricated electrode during 100 bending cycles (left) and 100 stretching cycles (right), showing negligible changes to prove mechanical stability. Like printed electrodes, we conducted an experimental study to validate the mechanical reliability of printed circuits (Figure 4D). Figure 4E shows measured resistance changes of a fabricated circuit during 100 bending cycles, showing no mechanical damage and signal fluctuation. An additional study in Figure 4F shows wirelessly measured 3-axis acceleration from the circuit obtained during 100 bending cycles, proving the device’s consistent performance in wireless connection and data acquisition.

### Demonstration of Detecting Physiological Signals using an All-in-One Bioelectronic Patch

**Figure 5:**
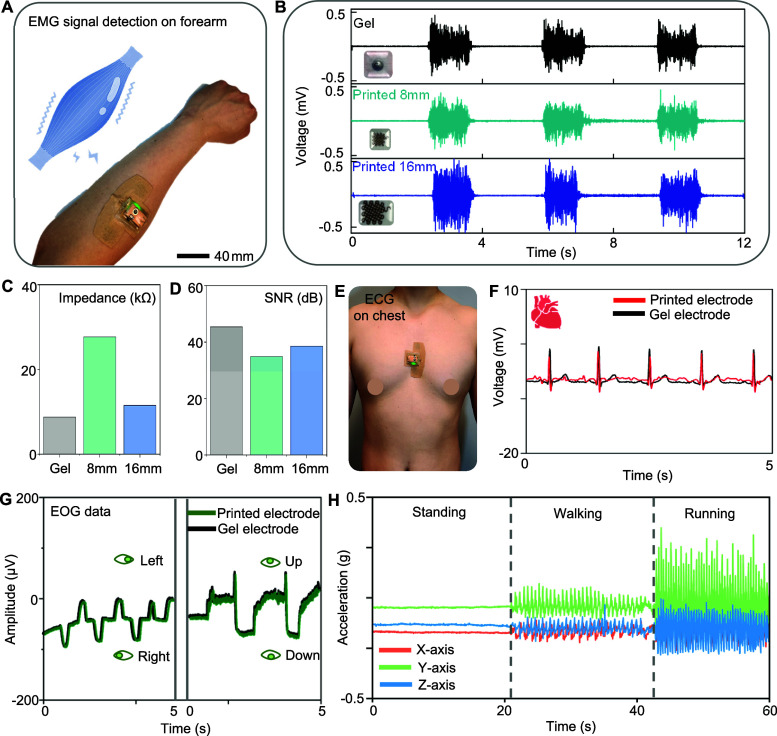
Demonstration of detecting physiological signals using an all-in-one bioelectronic patch. (A) EMG measurement setup of a wearable bioelectronic patch mounted on a subject’s forearm. (B) Experimental data of measured EMG signals on the forearm, comparing three types of electrodes, including a conventional gel electrode (top), a printed electrode (8 mm in the side) (middle), and a printed electrode (16 mm in the side). (C,D) Comparison of measured skin-electrode contact impedance (C) and SNR (D) between three electrodes. (E) ECG measurement setup of a wearable bioelectronic patch mounted on the chest. (F) Comparison of measured ECG data between a printed device and a conventional gel one, showing negligible difference. (G) Measured EOG data between two electrodes during the subject’s four types of eye movements, including left, right, up, and down. (H) Measured motions using an embedded accelerometer in the patch from the subject’s standing, walking, and running.

This study demonstrates the capability of an all-in-one printed bioelectronic patch by detecting various physiological signals in human subjects and comparing its performance with a clinical-grade commercial device. Figure 5A shows an integrated wireless bioelectronic system mounted on a subject’s forearm for measuring EMG signals. To validate the performance of printed electrodes, we compared EMG signals using three different electrodes: a commercial gel electrode (top), an 8 mm printed electrode (middle), and a 16 mm printed electrode (bottom graph). As summarized in Figure 5B, both printed electrodes consistently recorded high-quality EMG signals during three repetitive hand gestures, comparable to the commercial one. Figure 5C,D compare the skin impedance and signal-to-noise ratio (SNR) among three electrodes. As a result, the impedance of the 8 mm printed electrode (27.7 kΩ) is relatively higher than that of the gel electrode (8.7 kΩ). However, increasing the electrode size to 16 mm significantly reduces the impedance to 11.5 kΩ, approaching the value of the commercial one. Similarly, the 16 mm printed electrode shows a similar SNR (38.9) compared to the gel electrode (45.5). To demonstrate the advantages of the printed bioelectronic patch over traditional electrodes, we conducted comparative tests using our device and commercial Ag/AgCl gel electrodes. As shown in Figure S7, both devices were applied to the skin for 24 h to assess their biocompatibility and potential for skin irritation. The results indicate that the Ag/AgCl electrodes can cause noticeable skin irritation, while our printed bioelectronic patch does not have a side effect to the skin, highlighting its superior biocompatibility.^47^ Ag/AgCl gel electrodes also suffer from significant limitations in terms of reusability and durability. They are typically limited to single-use applications because the gel dries out, loses adhesion, and degrades in performance over time, especially when exposed to sweat, which can further accelerate the loss of conductivity and adhesion.^48−50^ Our printed bioelectronic patch shows better resilience to sweat exposure compared to Ag/AgCl electrodes, maintaining functionality for a longer period. While not completely immune to sweat-induced issues, accelerated tests in artificial sweat capture that our device experienced less severe performance degradation than Ag/AgCl electrodes, which are highly susceptible to deterioration in such conditions. The patch’s ability to be reused multiple times and remain effective for long-term monitoring demonstrates its durability and suitability for continuous health monitoring, especially where sweat and prolonged skin contact pose challenges for traditional gel electrodes. To demonstrate the versatility of printed wearable bioelectronics, we conducted additional tests with human subjects in measuring ECG, EOG, and motion signals. Figure 5E shows the placement of the chest for ECG measurement. For performance validation, our device was compared to a clinical-grade physiological monitoring system (BioRadio, Great Lakes Neuro-Technologies), which requires three gel electrodes. As summarized in Figure 5F, the printed bioelectronic patch captures the critical points of PQRST peaks in ECG data, demonstrating comparable ECG sensing performance to the clinical device. Additionally, we validated the EOG sensing performance of the printed patch against the gel electrode device, revealing that both systems capture clear EOG signals corresponding to the subject’s eye movements (Figures 5G and S8). Lastly, the wearable patch can measure motions using an embedded accelerometer from the subject’s standing, walking, and running on a treadmill (Figure 5H). Collectively, as summarized in Table 1, unlike prior work, the fabricated electronic system in this work demonstrates superior performance in detecting multiple physiological signals, validating the device’s versatility for potential use in many health monitoring and machine interface applications. Detailed material properties, printing parameters, and sintering methods for each ink in the device fabrication are summarized in Table 4.

**Table 4:** List of Ink Properties, AJP Printing, and Sintering Conditions

list of parameter	AgNP	CuNP	PEDOT:PSS	PI
solvent	xylene	DI-water	DI-water	*N*-methyl-2-pyrrolidone
viscosity (cP)	15	20	15–40	350
sheath rate (ccm)	30	30	25	20
atomization rate (ccm)	25	28	20	1000
exhaust rate (ccm)				1100
nozzle diameter (μm)	200	200	200	300
printing speed (mm/s)	10	10	10	10
platen temperature (°C)	70	50	70	80
sintering	thermal sintering at 250 °C for 1 h	photonic sintering at 3 kV, 3 ms	thermal sintering at 125 °C for 15 min	thermal sintering at 250 °C for 1 h

## Conclusions

This paper reports all-in-one integrated, flexible wearables and bioelectronics developed for detecting physiological signals in human subjects. This work illustrates the integration of Cu nanoparticles, Ag nanoparticles, PEDOT:PSS, and PI using AJP to fabricate ultrathin, multilayered electrodes, and circuits for wearable sensors and wireless systems. The addition of SAM significantly improves the adhesion of Cu ink, which in turn enhances Cu’s conductivity. Meanwhile, IPL sintering at room temperature reduces thermal damage. In addition, PEDOT:PSS ensures biocompatibility and prevents Cu-oxidation. The printed bioelectronic system effectively monitors various physiological signals, such as EMG, EOG, ECG, and motion data, making it suitable for a wide range of health monitoring and human–machine interface applications. Comprehensive mechanical testing confirms the flexibility and durability of the printed devices, demonstrating their suitability for long-term wear and dynamic use. Our future work will concentrate on harnessing the adaptability of AJP to investigate the use of different inks and materials for wider applications in bioelectronics. This approach will facilitate the creation of versatile, high-performance bioelectronic systems for a range of physiological monitoring and advanced health applications.

## Experimental Section

### Preparation of Inks

The process for preparing CuNP ink, as illustrated in Figure S2, begins with the creation of two solutions. The first solution involved mixing ethylene glycol (EG, Sigma-Aldrich), polyvinylpyrrolidone (PVP, Sigma-Aldrich), and sodium hypophosphite monohydrate (NaH_2_PO_2_·H_2_O, BEANTOWN CHEMICAL) at 90 °C and 350 rpm for 2 h. The second solution was prepared by mixing EG with copper(II) sulfate pentahydrate (CuSO_4_·5H_2_O, Sigma-Aldrich) at 90 °C and 350 rpm for 30 min. The two solutions were then combined and maintained at 90 °C for 3 min. The mixture was cooled, and the particles were dispersed using a vortex mixer and sonication. The resulting suspension was centrifuged at 6000 rpm for 10 min to isolate the CuNPs. The collected CuNPs were washed with acetone and ethanol and separated via centrifugation. Finally, the purified CuNPs were dispersed in DI water to the desired concentration. The preparation of Ag and PI inks followed previously reported methods.^1^ Ag nanoparticle ink (Ag40XL, UT Dots) was mixed with xylene (*m*-xylene, Sigma-Aldrich) to achieve a 20% Ag concentration. PI ink was prepared by mixing a precursor (PI-2545, DuPont) with a solvent (1-methyl-2-pyrrolidinone; NMP, Sigma-Aldrich) in a 4:1 ratio. Additionally, PEDOT:PSS ink (PH1000, MSE supplies) was acquired as a commercially available ink.

### Self-Assembled Monolayers Treatment

To enhance the adhesion of printed CuNPs onto a coated-PI substrate, we employed (3-mercaptopropyl)trimethoxysilane (MPTS, Sigma-Aldrich) and (3-aminopropyl)trimethoxysilane (APTES, Sigma-Aldrich) as self-assembled monolayers (SAMs). Before applying the SAMs, the coated-PI substrate was treated with oxygen plasma (Cute, Femto Science) to introduce hydroxyl functional groups on the surface, facilitating subsequent bonding with MPTS or APTES. A 50 mM solution of either MPTS or APTES was then applied to the plasma-treated substrate and incubated at 30 °C for 30 min. After incubation, any residual SAM was removed by sonication in ethanol, followed by drying with an air gun to prepare the surface for subsequent processes.

### Intense Pulsed Light Sintering Optimization

The CuNP ink was printed using AJP (Aerosol Jet 200, Optomec). Subsequently, sintering was performed with IPL equipment (IPL-45 kW_2100, PSTEK Co.) using a xenon lamp (Heraeus). To identify the optimal sintering conditions, we measured the resistivity of Cu across a broad range of voltages and durations, as illustrated in Figure 2B. This approach allowed us to determine the optimal settings for achieving the lowest resistivity in the printed CuNPs.

### Artificial Sweat Preparation

Artificial sweat was prepared following the reference test method^45^ EN 1811:2011 by simply mixing NH4OH solution (134 mM), urea (10 mM), NaCl (27 mM), KCl (6.1 mM), Na2SO4 (0.41 mM), choline chloride (143 mM), l(+)-ascorbic acid (10.2 mM), d(+)-glucose (0.17 mM), and l(+)-lactate solution (188 mM) in distilled water. The pH of the solution was adjusted to 6.5 ± 0.1 using 0.1 M HCl. Small quantities of certain constituents, such as vitamins, nitrogenous substances, organic acids, carbohydrates, and a few ionic constituents, were omitted except for key chemicals.

### Fabrication of Electrodes and Circuits

As illustrated in Figure S9, the process began by spin-coating a sacrificial layer of PMMA on a glass slide at 1000 rpm for 30 s and baking it at 200 °C for 2 min. PI ink was then spin-coated on the PMMA/glass substrate and cured at 250 °C for 1 h. After curing, the PI substrate underwent SAM treatment to enhance adhesion. CuNPs ink was printed on the SAM-treated PI substrate using AJP, followed by sintering with an IPL system. PEDOT:PSS ink was then printed and cured on a hot plate at 125 °C for 15 min. The structure was subsequently cut using a femtosecond laser (OPTEC). Finally, the sacrificial PMMA layer was removed in an acetone bath, and the electrodes were transferred onto an elastomer substrate. The circuit fabrication process, shown in Figure S10, began with spin-coating a PMMA sacrificial layer on a glass slide, followed by spin-coating the first PI layer. Ag ink was printed using a 200 μm diameter nozzle and sintered with IPL under previously optimized conditions.^1^ A second PI ink layer was aligned and printed on top of the Ag layer, followed by the printing of the second Ag layer. After aligning and printing the third PI layer, CuNPs ink was printed on the structure. The entire assembly was then immersed in an acetone bath to remove the sacrificial PMMA layer. Finally, chips were mounted and electrically connected to the circuit (Figure S11).

### Material Characterization

A profilometer (Dektak 150, Veeco) was employed to measure each printed layer. Scanning electron microscopy combined with a focused ion beam (SEM; Hitachi SU 8230) provided microscopy images. The crystallographic and elemental structures were analyzed using X-ray diffraction (XRD; X’Pert PRO Alpha-1, Malvern Panalytical) and X-ray photoelectron spectroscopy (XPS; K-Alpha XPS, Thermo Fisher). Cyclic mechanical tests were conducted with a digital force gauge (M5–5, Mark-10) mounted on a motorized test stand (ESM303, Mark-10). The sheet resistance of CuNPs patterns was measured by a four-point probe (CMT-SR2000N, AIT), and resistivity was calculated based on the sheet resistance and thickness of the pattern. Adhesion between the PI substrate and CuNPs patterns was evaluated using scratch and cross-cut tape tests (ASTM D3359). Precise adhesive strength was measured with a 180° peel tester (MCT-2150, AND Korea), as detailed in previous research.

### Finite Element Analysis

Finite element analysis was conducted using ABAQUS software (Dassault Systèmes Simulia Corporation) to estimate the mechanical behavior of the printed device. The study focused on the mechanical reliability of the multilayered device under repetitive bending and stretching, simulating the mechanical environment of a forearm. The simulation used the following material properties: Young’s modulus (*E*) and Poisson’s ratio (*v*): *E*_PI_ = 2.5 GPa, *v*_PI_ = 0.34 for PI; *E*_Ag_ = 40 GPa, *v*_Ag_ = 0.37 for Ag; *E*_Cu_ = 110 GPa, *v*_Cu_ = 0.34 for Cu; *E*_PEDOT:PSS_ = 2.8 GPa, *v*_PEDOT:PSS_ = 0.33 for PEDOT:PSS.

### Human Subject Study

Two healthy volunteers participated in this experiment. The Georgia Tech IRB approved the experimental protocol (#22289). Each participant was informed of the detailed experimental protocol before participating.

## Supporting Information Available

Contains additional figures and analyses to supplement the main text. Included are XPS analysis of CuNP ink (Figure S1), schematic illustration of the CuNP ink preparation process (Figure S2), images of AJP-printed and IPL-sintered CuNP surfaces before and after soldering (Figure S3), zeta potential comparison for CuNP with and without PVP (Figure S4), oxidation comparison in printed Cu electrodes with PEDOT:PSS capping (Figure S5), XPS oxidation analysis of Cu in artificial sweat (Figure S6), skin irritation test with Ag/AgCl and custom electrodes (Figure S7), EOG measurement setup with a commercial device (Figure S8), electrode and circuit fabrication schematics (Figures S9 and S10), and a schematic diagram of the printed circuit board (Figure S11) (PDF)
