# Supplementary material for: Printed Nanomaterials for All-in-One Integrated Flexible Wearables and Bioelectronics
Source: ACS Appl Mater Interfaces. 2024 Nov 25;16(49):68016–26. doi: 10.1021/acsami.4c17939 (PMC11647766; doi:10.1021/acsami.4c17939)
Supplement: Supplementary file 1 — am4c17939_si_001.pdf [file am4c17939_si_001.pdf]

Supporting Information

Printed Nanomaterials for All-in-One Integrated Flexible Wearables and Bioelectronics

Youngjin Kwon[a,b,†], *Jongsu Kim*[a,c,†], *Hojoong Kim*[c,d], *Tae Woog Kang*[a,c], *Jimin Lee*[a,c], *Seung Soon Jang*[a,b,*], *Yongkuk Lee*[e,*], *Woon-Hong Yeo*[a,c,d,f,g,*]

[a] Wearable Intelligent Systems and Healthcare Center (WISH Center), Institute for Matter and Systems, Georgia Institute of Technology, Atlanta, GA 30332, USA
[b] School of Materials Science and Engineering, Georgia Institute of Technology, Atlanta, GA 30332, USA
[c] George W. Woodruff School of Mechanical Engineering, Georgia Institute of Technology, Atlanta, GA 30332, USA
[d] Korea KIAT-Georgia Tech Semiconductor Electronics Center (K-GTSEC), Georgia Institute of Technology, Atlanta, GA 30332, USA
[e] Department of Biomedical Engineering, Wichita State University, Wichita, KS 67260, USA
[f] Wallace H. Coulter Department of Biomedical Engineering, Georgia Institute of Technology and Emory University School of Medicine, Atlanta, GA 30332, USA
[g] Parker H. Petit Institute for Bioengineering and Biosciences, Georgia Institute of Technology, Atlanta, GA 30332, USA
[†]Equal contributions to this paper
*Corresponding authors: Woon-Hong Yeo: whyeo@gatech.edu, Yongkuk Lee: yongkuk.lee@wichita.edu and Seung Soon Jang: seungsoon.jang@mse.gatech.edu

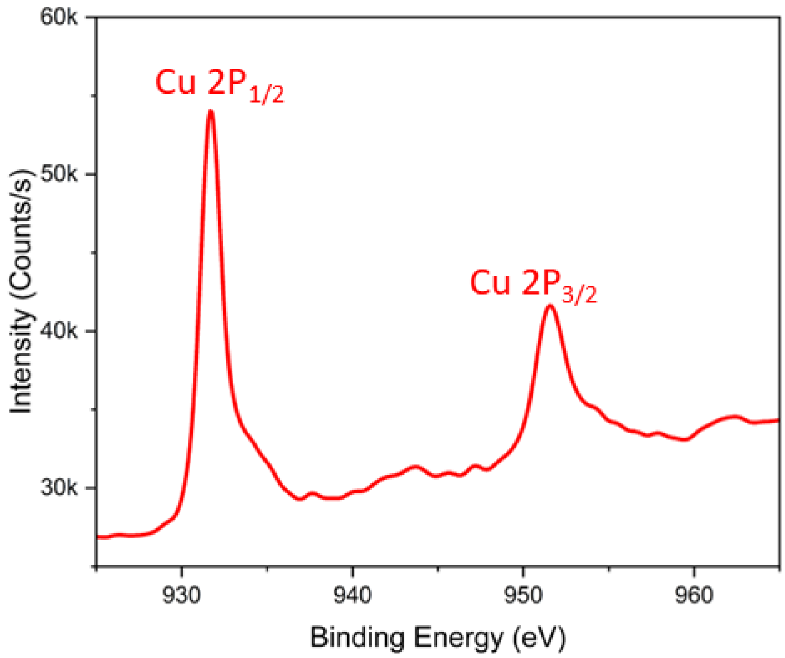

Figure S1. XPS analysis of the CuNP ink shows the elemental composition of the particles, with no significant signs of oxidation detected.

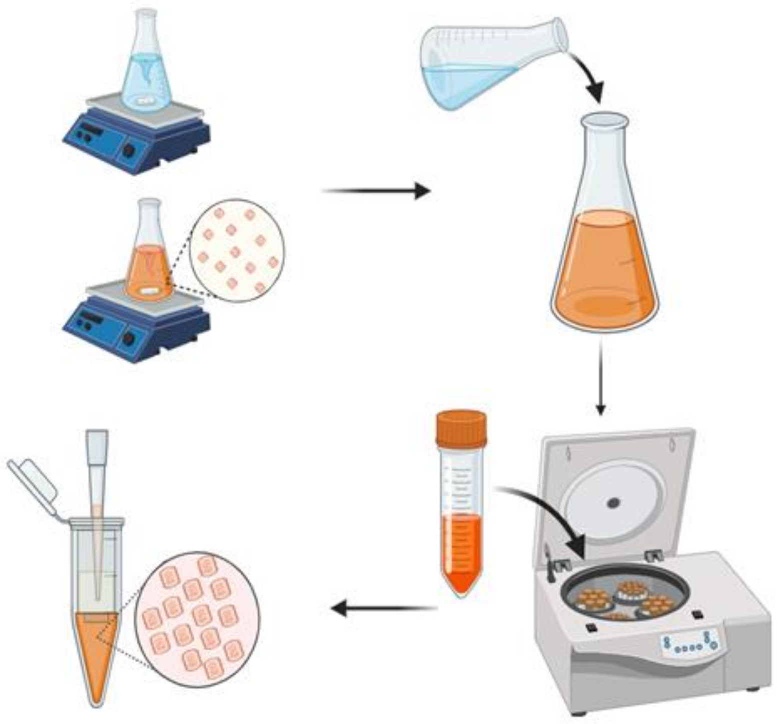

Figure S2. Schematic illustration of the process for preparing CuNP ink (created in BioRender, https://BioRender.com/q18w295).

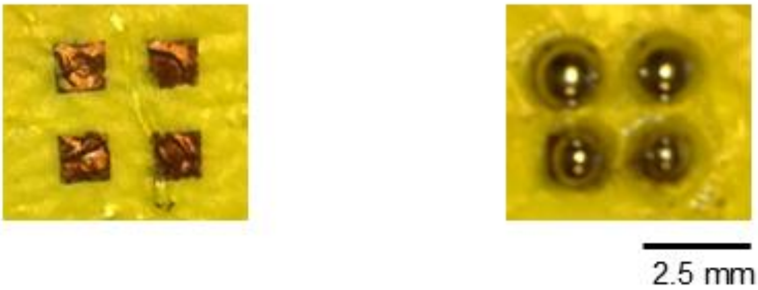

Figure S3. Images showing the surface of AJP-printed and IPL-sintered CuNP before and after soldering.

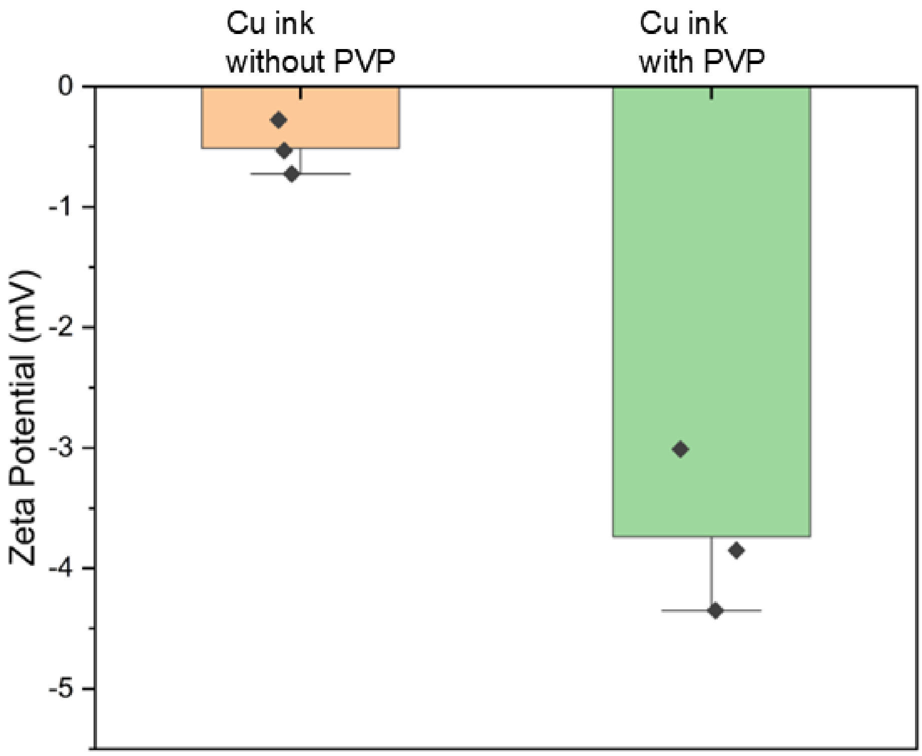

Figure S4. Comparison of zeta potential for CuNP with and without PVP.

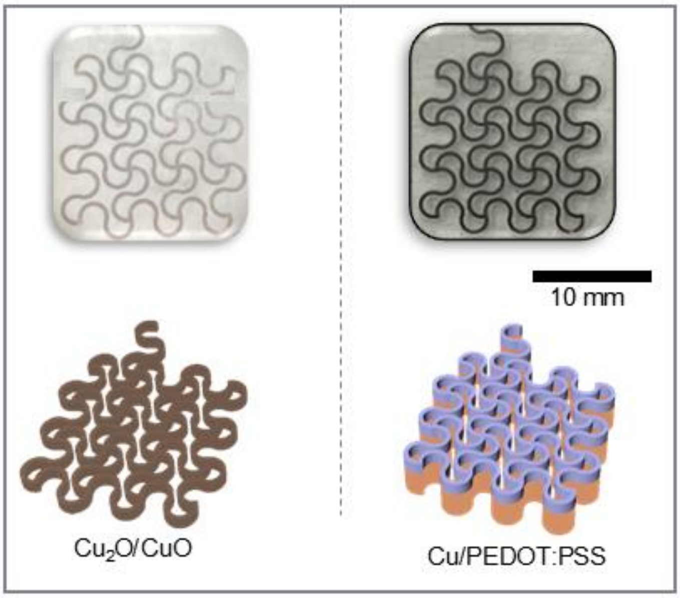

Figure S5. Comparison of oxidation in printed Cu electrodes with PEDOT:PSS capping.

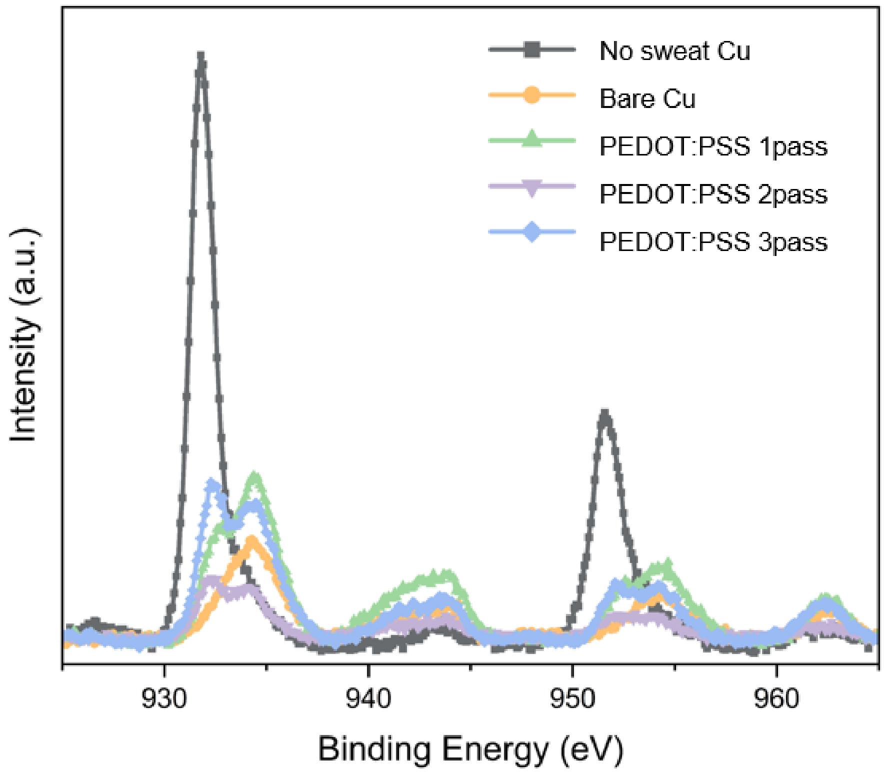

Figure S6. XPS analysis comparing the oxidation rates of untreated Cu, Cu after immersion in artificial sweat for 12 hours, and Cu with one, two, and three layers of PEDOT:PSS after immersion in artificial sweat for 12 hours.

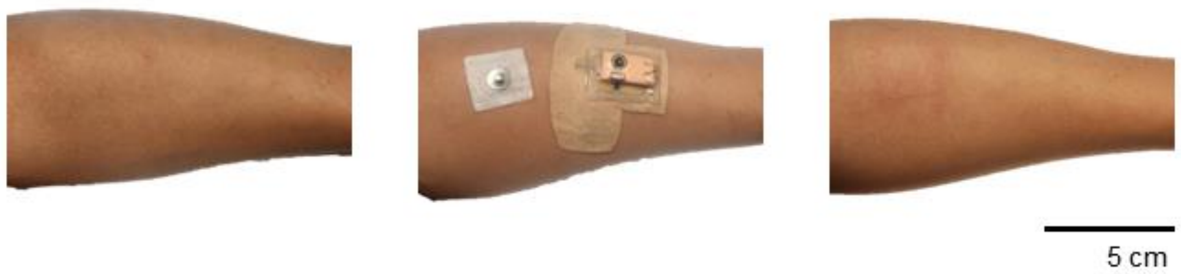

Figure S7. The irritation test shows before, during, and after 24 hours of application of commercial Ag/AgCl electrodes and our device on the skin.

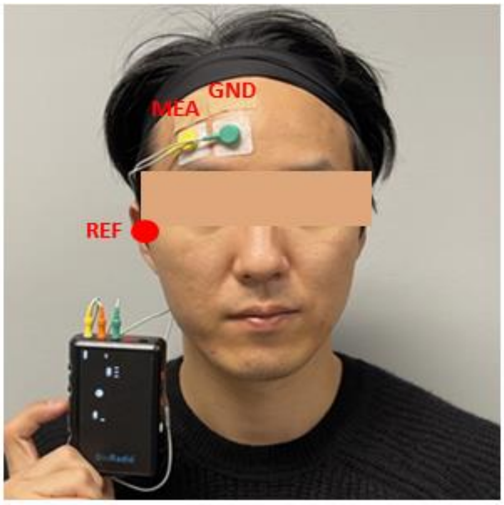

Figure S8. Photo of a subject who measures EOG using a commercial device.

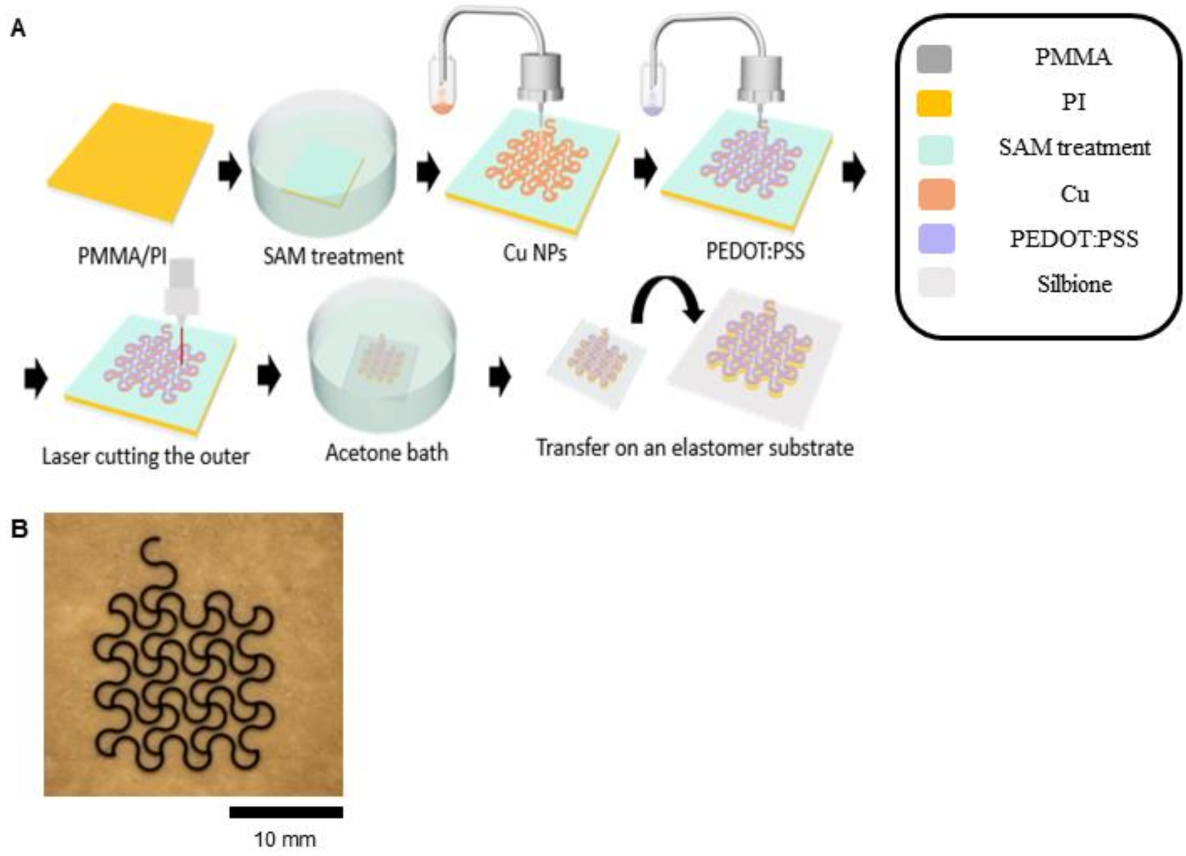

Figure S9. A) Schematic representation of the electrode fabrication process and B) Photo of a printed electrode.

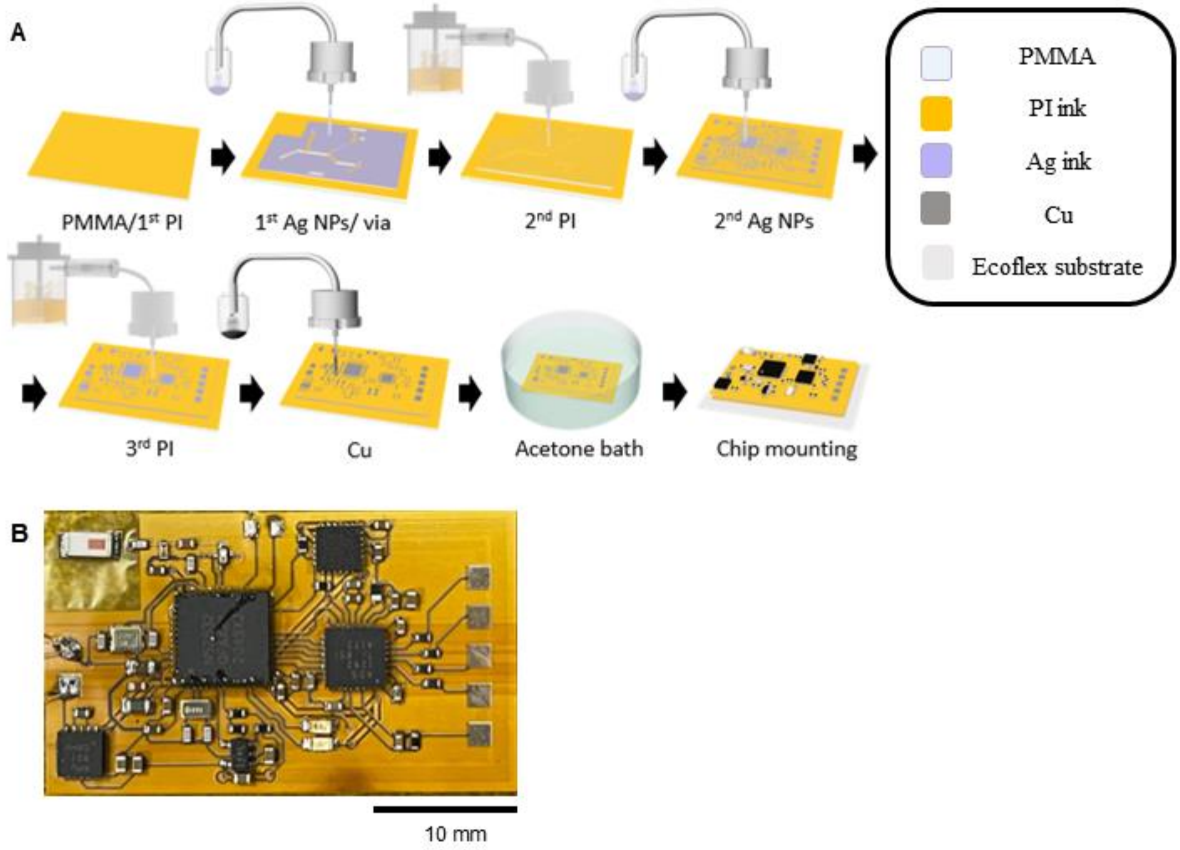

Figure S10. A) Schematic illustration of the circuit fabrication process and B) Photo of a printed circuit.

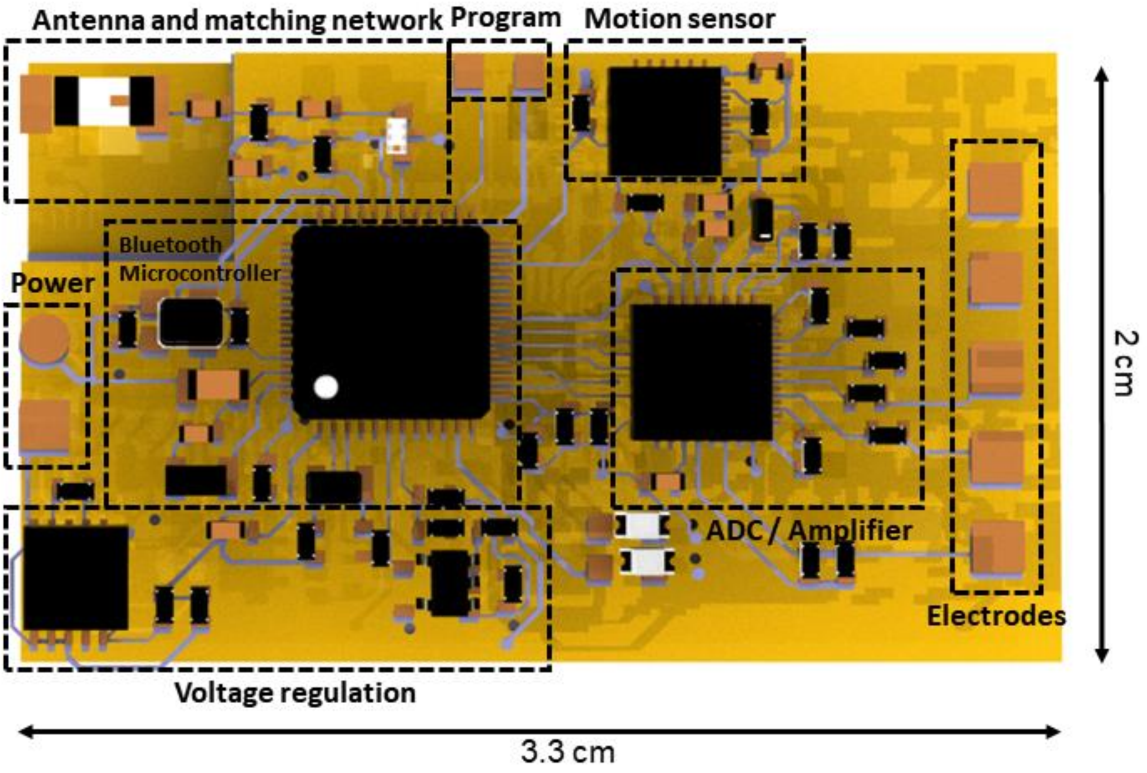

Figure S11. Schematic diagram of the printed circuit board.
